# Neutrophil-to-lymphocyte ratio as a predictor of prognosis in patients with spontaneous intracerebral hemorrhage: a systematic review and meta-analysis

**DOI:** 10.3389/fneur.2025.1553263

**Published:** 2025-03-21

**Authors:** Lixia Guo, Xudong Cao, Luyao Chang, Huandong Liu

**Affiliations:** ^1^Department of Medical College, Tibet University, Lhasa, China; ^2^Department of Neurosurgery, Tibet Autonomous Region People's Hospital, Lhasa, China

**Keywords:** neutrophil-to-lymphocyte ratio, intracerebral hemorrhage, prognosis, meta-analysis, systematic review

## Abstract

**Objective:**

To evaluate the predictive value of the neutrophil-to-lymphocyte ratio (NLR) for prognosis spontaneous intracerebral hemorrhage (ICH) patients.

**Methods:**

PubMed, EMBASE, Cochrane Library, Web of Science were used for screening literature on NLR predicting ICH prognosis from database up to January 2024. Case–control or cohort studies that provided statistical analysis data on NLR predicting ICH prognosis were included. Data were combined using odds ratio (OR) and standard mean differences (SMD) for categorical variables and continuous variables, respectively. Meta-analysis, subgroup analyses, and sensitivity analyses were performed by Review Manager 5.4 and Stata 15.0.

**Results:**

Meta-analysis of 21 studies with a total of 7,176 patients confirmed that NLR has a significant predictive value for mortality (SMD: 0.80, 95% CI: 0.58–1.02; OR: 1.10, 95% CI: 1.04–1.17) and neurological function outcomes (SMD: 0.66, 95% CI: 0.50–0.81; OR: 1.29, 95% CI: 1.17–1.41). NLR also significantly predicted the occurrence of stroke-associated pneumonia (SAP) (SMD: 0.54, 95% CI: 0.21–0.87). Subgroup analysis suggested that NLR had good predictive value for mortality in ICH patients aged ≥60 years, with hematoma volume > 15 mL, and NLR cut-off >7.5, and for neurological function in ICH patients, Asian patients, and those with NLR cut-off >7.5. The stability of the results was confirmed by sensitivity analysis.

**Conclusion:**

NLR can significantly predict mortality, neurological function outcomes, and SAP occurrence in ICH patients. NLR cut-off >7.5 has good predictive value for both mortality and neurological function in ICH patients. Considering the limitations of this study, such as small sample size and potential heterogeneity, prospective studies with larger sample sizes are needed to confirm the findings of this article.

**Systematic review registration:**

https://www.crd.york.ac.uk/PROSPERO/, identifier CRD42024544506.

## 1 Introduction

Spontaneous intracerebral hemorrhage (ICH) represents 10–30% of all stroke cases and is associated with elevated rates of incidence, mortality, and disability (1). A survey of 480,687 participants from 155 urban and rural areas in 31 Chinese provinces showed that the incidence of spontaneous ICH was 23.8% (2). The 30-day case fatality rate after ICH onset can reach 40% (3). Only about 20% of patients can recover to live independently after 6 months, placing a heavy burden on families and society (4). Currently, there are few effective treatments for spontaneous ICH.

Various prognostic scores have been developed to predict ICH outcomes. The most commonly used prognostic factors include patient age, GCS score at admission, hematoma location and volume, and whether the hematoma extends into the ventricles. However, existing scores have limitations and cannot accurately predict outcomes (5–7). According to clinical and experimental findings, increased WBC and ANC levels, along with reduced ALC, indicate a heightened risk of secondary brain injury and are linked to poor short-term neurological outcomes following ICH (8, 9). The inflammatory response plays a crucial role as one of the main pathological mechanisms responsible for secondary brain injury in ICH patients (10). In recent years, the neutrophil-to-lymphocyte ratio (NLR), a novel, easily obtainable, and economical biomarker of systemic inflammation, has been shown to have prognostic value in various diseases, including stroke. Studies have found that NLR is associated with outcomes of ICH and cerebral ischemia (11, 12). Elevated NLR indicates enhanced neutrophil-induced inflammatory response and decreased lymphocyte-mediated anti-inflammatory response (13). The inflammatory response becomes more pronounced with higher NLR values. Wang et al. (14) reported 224 patients with ICH symptoms within 24 h who were admitted to the emergency department of Shanghai Jiading District Central Hospital in China over 2 years. The NLR, assessed on the morning of the second day following admission, was significantly elevated in patients who died compared to those who survived and served as an independent predictor of mortality at 30 days. Tao et al. (15) conducted a retrospective analysis of patients with spontaneous ICH who were admitted to Sichuan University’s West China Hospital between July 2010 and January 2013. The modified Rankin Scale was used by staff who were unaware of the laboratory results to evaluate clinical outcomes 90 days after the initial assessment. Clinical outcomes were evaluated 90 days after the initial assessment using the modified Rankin Scale by personnel blinded to the laboratory data. Ninety days following the initial evaluation, staff members who were unaware of the laboratory results used the modified Rankin Scale to assess clinical outcomes. Other studies have shown that elevated NLR after ICH is not significantly associated with poor prognosis (16–18).

The prognostic value of the neutrophil-to-lymphocyte ratio (NLR) is being increasingly recognized in a range of diseases, such as cardiovascular disorders and ischemic stroke (19, 20). The neutrophil-to-lymphocyte ratio (NLR) is gaining recognition as a valuable prognostic marker for various medical conditions, including cardiovascular diseases and ischemic stroke. Wang et al. (14) found that NLR at admission had no impact on 1-month mortality, while Lattanzi et al. (12) detected a negative correlation between NLR at admission and 3-month prognosis. Currently, studies on NLR predicting ICH prognosis are mainly retrospective, with no clear consensus and no evaluation of summarized data from previous studies. Moreover, the conclusions of these studies may be influenced by confounding factors. This study aims to summarize the prognostic value of NLR in ICH through meta-analysis, guiding early clinical diagnosis and intervention to improve prognosis.

## 2 Methods

### 2.1 Search strategy

This evidence-based analysis followed the Preferred Reporting Items for Systematic Reviews and Meta-Analyses (PRISMA) 2020 statement (21) and was prospectively registered in PROSPERO (CRD42024544506). The PRISMA 2020 checklist can be found in the Supplementary Table. A comprehensive search for relevant studies on the prognostic value of NLR in ICH was conducted in various English databases, such as PubMed, EMBASE, Cochrane Library, and Web of Science, covering the period from the establishment of each database to January 2024. The English search terms were “Neutrophils,” “Lymphocytes,” “ratio,” and “Intracranial Hemorrhages.” The complete search strategies for 4 databases are provided in the Supplementary Table 1. Furthermore, a manual review of the reference lists of all qualifying studies was conducted. The included studies were independently searched and assessed by two researchers, and any disagreements that arose during the literature search process were settled through mutual agreement.

### 2.2 Inclusion criteria

1. Published literature on NLR predicting ICH prognosis;
2. Case–control or cohort studies;
3. Cases were divided into good and poor prognosis groups, or high and low NLR groups;
4. ICH was confirmed by CT;
5. Included studies provided statistical analysis data on NLR predicting ICH prognosis, including weighted mean difference (WMD), standardized mean difference (SMD), and odds ratio (OR).

### 2.3 Exclusion criteria

1. Inappropriate study design, such as lack of a control group;
2. Non-English literature;
3. Lack of clear diagnostic criteria for ICH and prognosis;
4. Studies involving children;
5. Duplicate publications, poor quality, or incomplete data;
6. Reviews, case reports, commentaries, or conference papers.

### 2.4 Literature screening and data extraction

Moreover, two researchers independently searched for and evaluated the qualifying studies, with any disagreements during the literature search process being resolved through consensus. A manual examination of the reference lists from all included studies was also carried out. For studies with missing data, efforts were made to contact the original authors for supplementation. Extracted data included: (1) Basic information of the included studies: author name, publication year, country; (2) Basic characteristics of the study subjects: sample size, age, blood pressure; and (3) OR/RR values and 95% CI were extracted from multivariate analyses with NLR as a binary variable, and SMD and 95% CI were extracted from multivariate analyses with NLR as a continuous variable.

### 2.5 Quality assessment

The Newcastle-Ottawa Scale (NOS) was used to assess the quality of each study. The NOS uses three dimensions: “selection,” “comparability,” and “outcome,” with a total of 9 items and a score range of 0–9. A score of ≥6 was considered high quality.

### 2.6 Statistical analysis

Review Manager 5.4 software was used for meta-analysis. For binary variables (qualitative data), the pooled odds ratio (OR) and 95% confidence interval (95% CI) were calculated. For continuous variables (quantitative data), 95% CI and mean difference (MD) or standardized mean difference (SMD) were used for analysis. To assess heterogeneity across the included studies, Egger’s test was performed utilizing Stata software. When publication bias exists, the trimming and filling method is used to clarify whether the results are significantly affected by publication bias. A *p*-value exceeding 0.05 signified an absence of significant heterogeneity, whereas a *p*-value below 0.05 indicated the presence of substantial heterogeneity. The reliability of the pooled results was reflected by the consistency of the sensitivity analysis results, which were analyzed using Stata software. Subgroup analysis: This study performed subgroup analyses based on sample size, ICH type, region, follow-up time, age, hematoma volume, NLR threshold, and other factors for the outcome indicators of mortality (continuous), mortality (categorical), neurological function (continuous), neurological function (categorical), SAP (continuous), etc., to explore the stability of NLR’s predictive value for ICH and potential sources of heterogeneity.

## 3 Results

### 3.1 Literature search results

**Figure 1:**
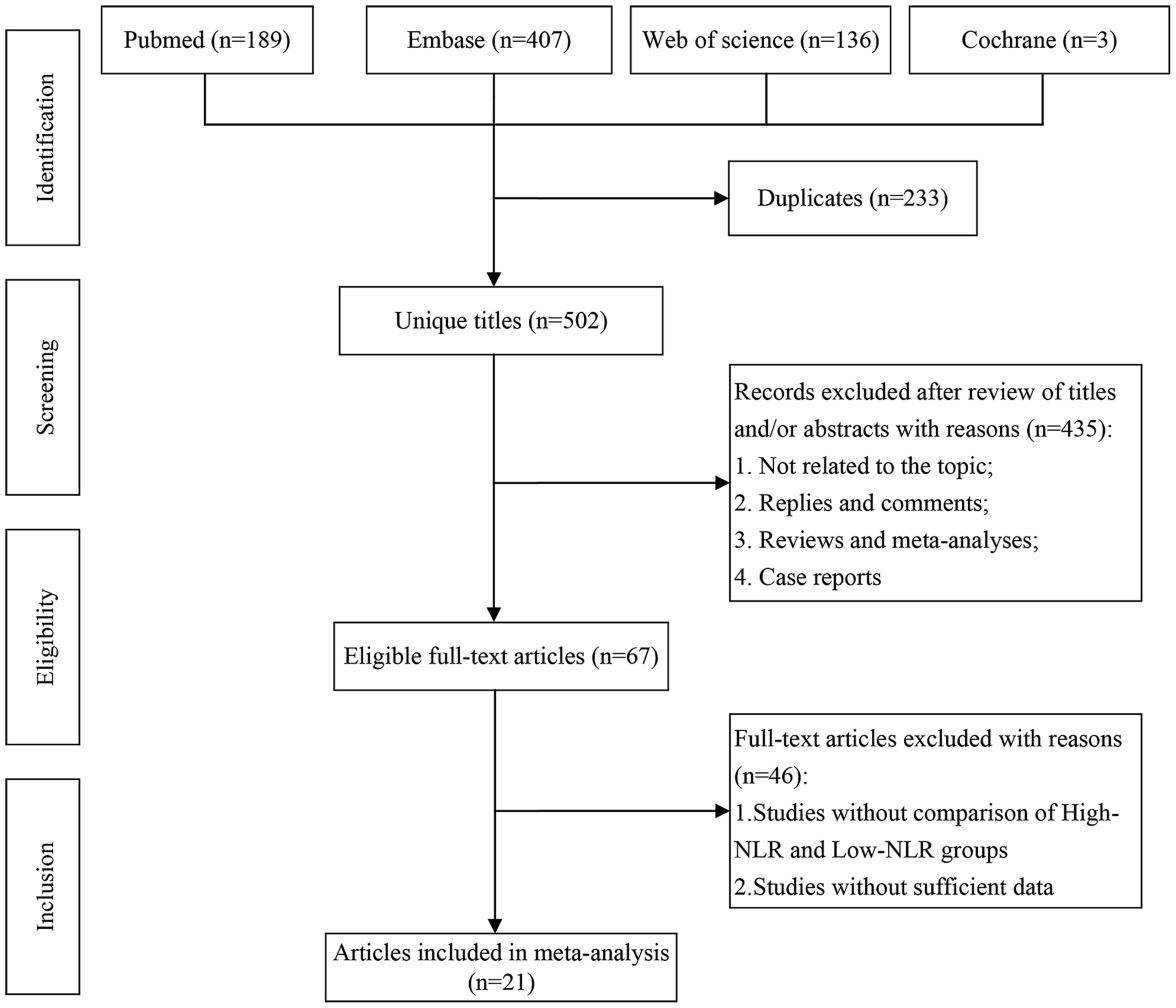
Literature screening flow chart.

Initially, 735 articles were identified from the search. Out of these, 233 were removed as they were duplicates. After browsing the titles and abstracts, 435 articles were further excluded based on the inclusion criteria. After full-text reading, 46 articles were excluded, leaving 21 articles (14, 15, 22–34) for analysis. The flow chart is shown in Figure 1.

### 3.2 Study characteristics

Study characteristics are presented in Table 1. This meta-analysis included a total of 7,176 patients, with 4,306 males and 2,870 females. The NLR cut-off values ranged from 5.50 to 14.77. The median NOS score of all studies was 7, ranging from 7 to 9, indicating that all included studies were of high quality. After literature search, 21 case–control and cohort studies were finally included. When grouped by mortality, there were a total of 765 deaths and 2,289 survivors. When grouped by neurological function prognosis, there were 2,192 cases in the poor prognosis group and 1814 cases in the good prognosis group; when grouped by whether SAP occurred, there were 615 cases with SAP and 305 cases without SAP. Mortality was assessed by reviewing electronic medical records and telephone follow-up. The evaluation of patient prognosis primarily utilized two scales: the Glasgow Outcome Scale (GOS) and the modified version of the Rankin Scale (mRS). Twelve comparable factors were included, such as gender, age, hematoma volume, and NLR threshold. The 21 studies were mainly from Asia and Europe, and all studies were evaluated using NOS, with scores ≥6. Detailed quality assessment results are provided in the Supplementary Table 2.

**Table 1:** Baseline characteristics of include studies.

Authors	Study period	Country	Study design	Patients (*n*)	Types of ICH	Duration of follow-up	Gender		Age	NLR cut-off
							Male	Female		
Tao, C. Y. 2016-I (15)	2010.7–2013.1	China	Retrospective cohort	336	ICH	90d	216	120	58.5 ± 13.0	6.62/6.28
Tao, C. Y. 2016-II (31)	2014.1–2015.12	China	Prospective cohort	247	aSAH	90d	88	159	55.9 ± 11.9	14.0
Wang, R. H. 2023 (29)	2019.5–2022.5	China	Retrospective cohort	320	ICH	7d	213	107	62.5 (51.3–73.0)	6.06
Menon, G. 2021 (36)	2015.1–2018.12	India	Retrospective cohort	851	ICH	90d	604	247	58.09 ± 12.85	8.2
Lin, M. Q. 2023 (35)	2010.1–2020.7	China	Retrospective cohort	1,132	ICH	7 d	790	342	59.09 ± 11.18	6.3
Pereira, M. 2023 (34)	2009.1–2018.12	Portugal	Retrospective cohort	130	ICH	30d	67	63	82.1 ± 4.8	
Wang, F. 2015 (14)	2012–2014	China	Retrospective cohort	224	ICH	30d	141	83	67.97 ± 13.75	7.35
Giede-Jeppe, A. 2018 (38)	2008–2012	Germany	Retrospective cohort	319	aSAH	90d	98	221		7.05
Radu, R. A. 2021 (32)	2018.7–2020.7	Romania	Retrospective cohort	201	ICH	30d	111	90	70 (61–79)	6.3
Lattanzi, S. 2018 (37)	2008.1–2017.3	Italy	Retrospective cohort	208	ICH	30d	132	76	66.7 (12.4)	
Wang, Z. G. 2019 (28)	2014.1–2017.1	China	Retrospective cohort	123	ICH		91	32	63.01 ± 10.34	6.49
Li, L. 2022 (35)	2001–2012	China	Retrospective cohort	1,000	ICH		546	454	68.05 ± 15.42	7.678
Lattanzi, S. 2016 (12)	2008.1–2015.9	Italy	Retrospective cohort	177	ICH	90d	63	114	67.1 (12.51)	
Volbers, B. 2018 (30)	2006.1–2014.1	Germany	Retrospective cohort	292	ICH	90d	161	131	70 (62–78)	
Zhang, F. 2019-I (25)	2010.2–2017.10	China	Retrospective cohort	107	ICH	30d	72	35	54.74 ± 12.04	7.04
Zhang, F. 2019-II (26)	2013.10–2017.5	China	Retrospective cohort	481	ICH	180d	350	131	61.09 ± 12.14	
Zhao, Y. 2022 (22)	2015.1–2021.6	China	Retrospective cohort	128	ICH	90d	88	40	60 (50.0–67.0)	12.35
Qiu, W. J. 2023 (33)	2018.1–2021.12	China	Retrospective cohort	321	aSAH	90d	140	181	≤61.5152 (67.86%) > 61.5 72 (32.14%)	14.77
Yang, W. 2021-I (27)	2018.1–2021.12	China	Retrospective cohort	431	ICH	90d	299	132	58.76 ± 12:92	9.32
Yang, W. 2021-II (27)	2018.10–2020.3	China	Retrospective cohort	251	ICH	90d	166	85	59.21 ± 13:57	10.04
Zhang, P. 2020 (24)	2015.1–2017.12	China	Retrospective cohort	178	aSAH	90d	62	116	57.64 ± 10.23	
Zhao, Y. 2023-I (23)	2019.1–2023.4	China	Retrospective cohort	200	ICH	90d	158	42	61 ± 16	
Zhao, Y. 2023-II (23)	2019.1–2023.4	China	Retrospective cohort	200	ICH	90d	158	42	61 ± 16	
Zhao, Y. 2023-III (23)	2019.1–2023.4	China	Retrospective cohort	200	ICH	90d	158	42	61 ± 16	

### 3.3 Meta-analysis results

Overall, the full-text comparison data of the 21 articles (14, 15, 22–34) included 6 prognostic outcome indicators: mortality, neurological function, SAP occurrence, post-stroke epilepsy, early hematoma expansion, and hematoma recurrence. Meta-analysis was performed on the extractable data of 3 indicators. Among the 3 prognostic outcomes, based on different statistical expressions, binary variables were grouped into “high NLR” and “low NLR,” while continuous variables were grouped into “survival,” “death,” “good prognosis,” “poor prognosis,” “SAP occurrence,” or “no SAP occurrence.” Subgroup analyses were conducted for different influencing factors within the same outcome.

#### 3.3.1 Predictive value of NLR for mortality

**Figure 2:**
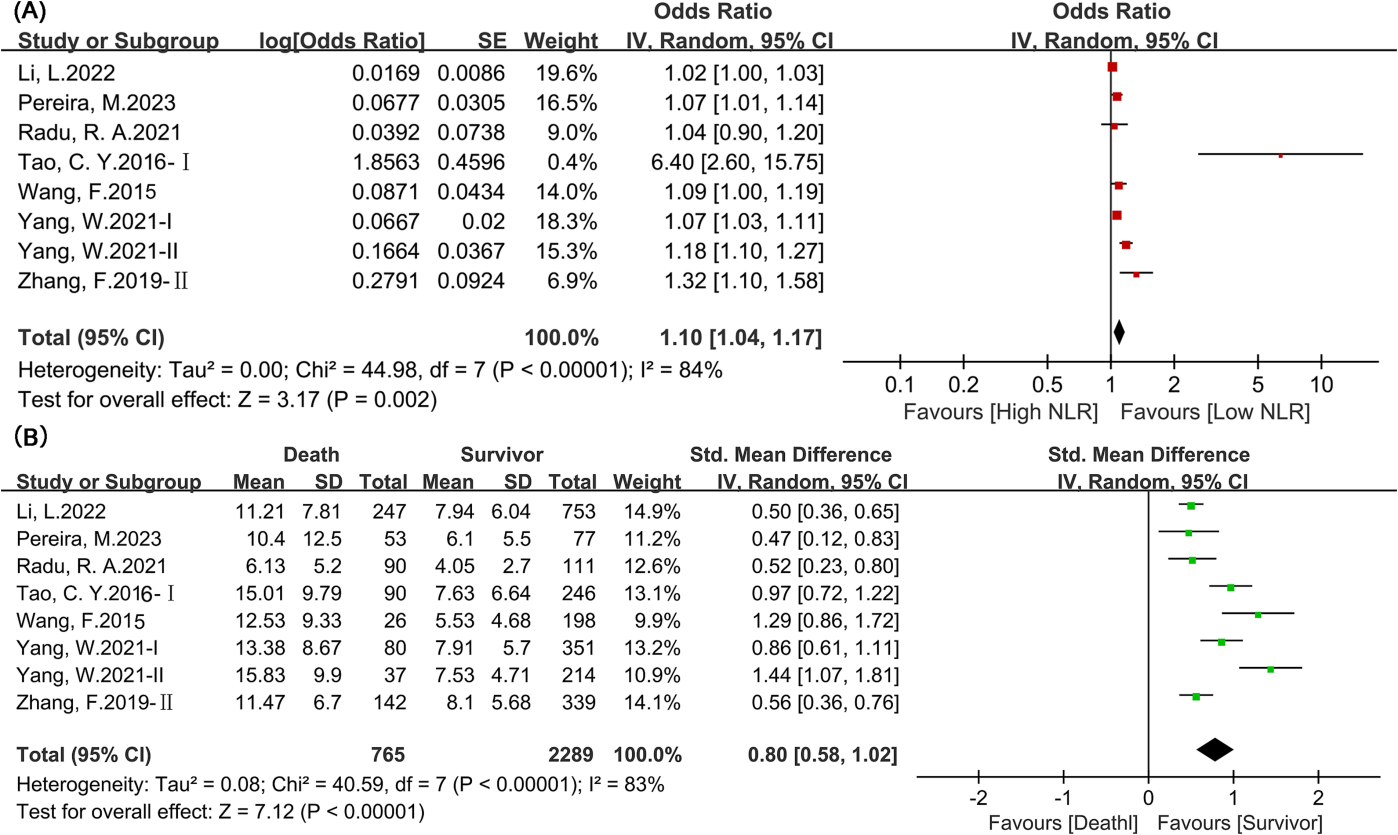
Mortality forest map. **(A)** Categorical variable; **(B)** Continuous variable.

Meta-analysis results for categorical variables: This study included 7 articles (14, 15, 26, 27, 32, 34, 35) for the meta-analysis of mortality (categorical) variables. The forest plot (Figure 2A) showed that higher NLR was significantly associated with increased patient mortality (OR: 1.10, 95% CI: 1.04–1.17). Significant heterogeneity was also present (I^2^ = 84%). Additionally, Egger’s test *p* = 0.005, and the funnel plot suggested publication bias. The trimming and filling method found that the predictive value of NLR for mortality was not significantly affected by publication bias (OR: 1.09, 95% CI: 1.02–1.17) (Supplementary Figure 1).

Results for continuous variables: This study included 7 articles (14, 15, 26, 27, 32, 34, 35) with a total of 3,054 patients for the meta-analysis of mortality (continuous) variables. The forest plot (Figure 2B) showed that NLR levels were significantly higher in deceased patients than in surviving patients (SMD: 0.80, 95% CI: 0.58–1.02). Significant heterogeneity was also present (I^2^ = 83%). Egger’s test *p* = 0.06, and the funnel plot suggested no publication bias.

#### 3.3.2 Predictive value of NLR for neurological function prognosis

**Figure 3:**
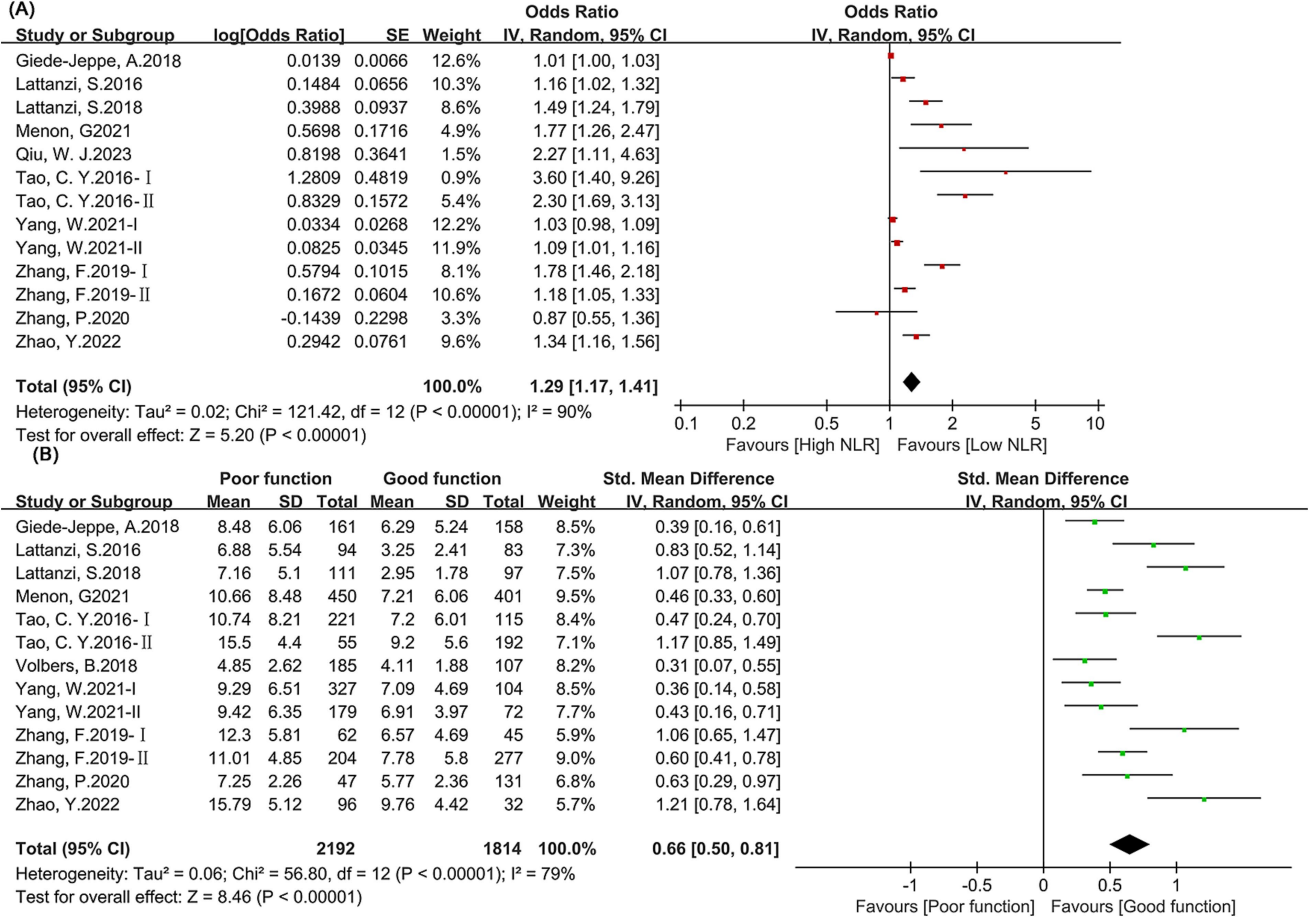
Forest map of neural function. **(A)** Categorical variable; **(B)** Continuous variable.

This study included 12 articles (12, 15, 22, 24–27, 31, 33, 36–38) for the meta-analysis of neurological function outcome (categorical) variables. The forest plot (Figure 3A) showed that higher NLR was significantly associated with poor neurological function outcomes (OR: 1.29, 95% CI: 1.17–1.41). Significant heterogeneity was also present (I^2^ = 90%). Egger’s test *p* = 0.54, and the funnel plot suggested no publication bias.

This study included 12 articles (12, 15, 22, 24–27, 30, 31, 36–38) with a total of 4,006 patients for the meta-analysis of neurological function outcome (continuous) variables. The forest plot (Figure 3B) showed that NLR levels were significantly higher in patients with poor neurological function than in those with good neurological function (SMD: 0.66, 95% CI: 0.50–0.81). Egger’s test *p* = 0.02, and the funnel plot suggested publication bias. The trimming and filling method found that the predictive value of NLR for neurological function prognosis was not significantly affected by publication bias (SMD: 0.62, 95% CI: 0.47–0.78) (Supplementary Figure 2). Subgroup analysis found that the results were stable across subgroups when NLR predicted mortality, with no significant differences.

#### 3.3.3 Predictive value of NLR for SAP

**Figure 4:**
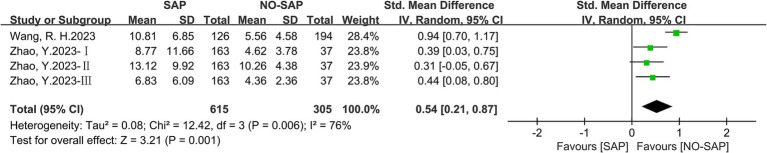
SAP forest map.

This study included 2 articles (23, 29) with a total of 920 patients for the meta-analysis of SAP occurrence (continuous) variables. The forest plot (Figure 4) showed that NLR levels were significantly higher in patients with SAP than in those without SAP (SMD: 0.54, 95% CI: 0.21–0.87). Egger’s test *p* = 0.01, and the funnel plot suggested publication bias. The trimming and filling method found that the predictive value of NLR for SAP was not significantly affected by publication bias (SMD: 0.54, 95% CI: 0.21–0.88) (Supplementary Figure 3).

### 3.4 Sensitivity analysis

This study found that NLR showed stability in predicting mortality (continuous), mortality (categorical), neurological function outcome (continuous), neurological function outcome (categorical), SAP occurrence (continuous), and other prognostic outcome indicators in ICH patients. The results remained stable and were not affected by extreme values when any single study was removed. The sensitivity analysis results are shown in Supplementary Figures 4–8.

### 3.5 Subgroup analysis

Subgroup analysis of mortality (categorical) found that NLR remained significant in predicting mortality in the age > 60 group, hematoma volume ≥ 15 mL group, and NLR threshold ≥7.5 group. However, NLR’s predictive value for mortality was not significant in the age ≤ 60 group (*p* = 0.05; I^2^ = 90%), hematoma volume < 15 mL group (*p* = 0.05; I^2^ = 0%), and NLR threshold <7.5 group (*p* = 0.14; I^2^ = 87%). Subgroup analysis of neurological function (categorical) suggested that NLR’s predictive value for neurological function outcome was not significant in patients with subarachnoid hemorrhage (*p* = 0.18; I^2^ = 91%), European region (*p* = 0.1; I^2^ = 90%), and NLR threshold <7.5 (*p* = 0.09; I^2^ = 95%). However, NLR significantly predicted neurological function outcomes in patients with ICH, Asian region, and NLR threshold >7.5. Detailed subgroup analysis data are provided in Table 2.

**Table 2:** The subgroup results of meta-analysis.

Subgroup	Mortality (continuous)				Mortality rate (categorical variables)				Neural function (continuous)				Neural function (categorical variables)			
	Study	SMD	*p* value	*I* ^2^	Study	OR [95%CI]	*p* value	*I* ^2^	Study	SMD	*p* value	*I* ^2^	Study	OR [95%CI]	*p* value	*I* ^2^
Total	8	0.80 [0.58, 1.02]	*p* < 0.00001	83%	8	1.10 [1.04, 1.17]	0.002	84%	13	0.66 [0.50, 0.81]	*p* < 0.00001	79%	13	1.29 [1.17, 1.41]	*p* < 0.00001	90%
Sample size																
>250	5	0.83 [0.56, 1.11]	*p* < 0.00001	87%	5	1.13 [1.03, 1.24]	0.007	91%	6	0.98 [0.81, 1.16]	*p* < 0.00001	37%	6	1.10 [1.02, 1.18]	0.02	82%
≤250	3	0.74 [0.28, 1.20]	0.002	81%	3	1.07 [1.02, 1.12]	0.003	0%	7	0.45 [0.37, 0.52]	*p* < 0.00001	0%	7	1.48 [1.22, 1.79]	*p* < 0.00001	80%
Types of cerebral hemorrhage																
ICH	8	0.80 [0.58, 1.02]	*p* < 0.00001	83%	8	1.10 [1.04, 1.17]	0.002	84%	10	0.64 [0.47, 0.80]	*p* < 0.00001	78%	9	1.30 [1.16, 1.46]	*p* < 0.00001	87%
aASH									3	0.72 [0.25, 1.19]	0.003	87%	4	1.42 [0.86, 2.34]	0.18	91%
Region																
Asia	6	0.90 [0.63, 1.17]	*p* < 0.00001	87%	6	1.12 [1.04, 1.21]	0.004	89%	9	0.67 [0.49, 0.84]	*p* < 0.00001	78%	10	1.38 [1.19, 1.60]	*p* < 0.00001	88%
Europe	2	0.50 [0.28, 0.72]	*p* < 0.00001	0%	2	1.07 [1.01, 1.13]	0.02	0%	4	0.64 [0.29, 0.99]	0.0003	86%	3	1.18 [0.97, 1.45]	0.1	90%
Categorical variables																
>30d	4	0.65 [0.37, 0.94]	*p* < 0.00001	75%	4	1.22 [1.05, 1.43]	0.009	88%	10	0.54 [0.41, 0.67]	*p* < 0.00001	69%	10	1.18 [1.08, 1.28]	0.0003	86%
≤30d	4	0.93 [0.61, 1.25]	*p* < 0.00001	84%	4	1.04 [1.00, 1.08]	0.03	39%	3	1.10 [0.89, 1.31]	*p* < 0.00001	0%	3	1.51 [1.29, 1.78]	*p* < 0.00001	60%
Mean age																
>60	5	0.62 [0.42, 0.82]	*p* < 0.00001	67%	5	1.07 [1.01, 1.13]	0.02	68%	5	0.78 [0.48, 1.07]	*p* < 0.00001	83%	4	1.27 [1.14, 1.41]	*p* < 0.00001	54%
≤60	3	1.06 [0.76, 1.36]	*p* < 0.00001	70%	3	1.19 [1.00, 1.42]	0.05	90%	7	0.62 [0.42, 0.82]	*p* < 0.00001	77%	8	1.45 [1.20, 1.75]	0.0001	90%
Mean volume																
≥15 mL	5	0.85 [0.56, 1.13]	*p* < 0.00001	82%	5	1.16 [1.05, 1.28]	0.003	84%	7	0.50 [0.36, 0.64]	*p* < 0.00001	62%	6	1.20 [1.07, 1.35]	0.002	82%
<15 mL	2	0.89 [0.13, 1.64]	0.02	89%	2	1.08 [1.00, 1.16]	0.05	0%	3	0.98 [0.79, 1.17]	*p* < 0.00001	0%	3	1.44 [1.12, 1.87]	0.005	86%
NLR cut-off																
≥7.5	/	/	/	/	3	1.08 [1.01, 1.16]	0.03	90%	/	/	/	/	6	1.36 [1.15, 1.61]	0.0003	89%
<7.5	/	/	/	/	3	1.25 [0.93, 1.69]	0.14	87%	/	/	/	/	3	1.60 [0.94, 2.73]	0.09	95%

## 4 Discussion

Inflammatory processes contribute to secondary brain damage following ICH. The NLR serves as a broad marker reflecting the systemic inflammatory response after a stroke event. We aimed to evaluate the predictive value of NLR for ICH prognosis. Wang et al. (29) studied 320 ICH patients and found that NLR could predict the occurrence of SAP and poor outcomes at discharge, and NLR might help early identification of severe SAP and predict ICU admission. Wang et al. (29) studied 224 ICH patients and found that the higher the NLR, the higher the mortality rate in ICH patients. NLR could be used to predict the 30-day prognosis of ICH patients. The study conducted by Lattanzi et al. (37) involved 177 patients with ICH. Their findings indicated that elevated neutrophil counts, diminished lymphocyte levels, and a higher NLR were associated with poorer neurological outcomes at the 3-month follow-up.

The findings of this research, considering both continuous and categorical variables, demonstrated that the NLR exhibited significant predictive capability for mortality outcomes. Additionally, this study found that NLR also had significant predictive value for neurological function outcomes and SAP. Furthermore, sensitivity analysis of the included indicators revealed that the statistical differences were stable for all outcome indicators and were not significantly affected by a single study. However, publication bias testing suggested significant publication bias for mortality (categorical), neurological function (continuous), and SAP (continuous) indicators, indicating that caution should be exercised when interpreting these indicators. Liu et al. (39) published a meta-analysis in 2019, concluding that high NLR was a predictor of severe disability and mortality in ICH patients in the short term, but not a predictor of in-hospital mortality. Guo et al. (40) published a meta-analysis in 2022, concluding that elevated NLR was an independent predictor of poor prognosis and delayed cerebral ischemia (DCI) occurrence in aSAH. The results of this study are generally consistent with the conclusions of previous meta-analyses, further confirming the predictive value of NLR for ICH prognosis.

Subgroup analysis of this study found that the predictive value of NLR for the prognosis of patients with cerebral hemorrhage was not significant in younger patients, and when the NLR cut-off was lower than 7.5, its predictive value was also not significant. Elderly patients often have immune senescence, which is manifested as a decrease in the number of lymphocytes and functional decline, and at the same time, the activity of neutrophils is enhanced, resulting in an increase in the baseline value of NLR (41). After cerebral hemorrhage, the inflammatory response of elderly patients is more likely to be out of control, and a high NLR may reflect more severe secondary brain damage (such as blood–brain barrier damage and release of neurotoxic mediators) (42). In young patients, the immune system has a strong compensatory ability, and NLR fluctuations may not be sufficient to trigger irreversible pathological processes, resulting in insignificant predictive efficacy (32). In addition, although subgroup analysis found that cut-off may affect the predictive value of NLR, there is still no conclusion on the best predictive value of NLR. Therefore, more prospective studies may be needed to consider setting multiple cut-offs to further verify the best predictive value of NLR and use it to clinically identify high-risk patients.

While the neutrophil-to-lymphocyte ratio (NLR) appears associated with unfavorable outcomes, particularly mortality following intracerebral hemorrhage (ICH), the underlying causal link requires further exploration. Several potential explanations for this relationship may exist. Firstly, the neutrophil-to-lymphocyte ratio (NLR) serves as a broad marker indicative of pro-inflammatory conditions and immunosuppression. Preclinical studies in animal models have demonstrated that inflammatory cells, mediators, and cytokines directly contribute to endothelial cell damage, neuronal death, and white matter injury, ultimately resulting in secondary brain insult (43). The presence of systemic inflammatory response syndrome can serve as a valuable indicator reflecting the clinical and radiological severity of stroke (44) exhibiting an inverse correlation with the prognosis in patients suffering from intracerebral hemorrhage (45). Moreover, ICH-induced immunosuppression can lead to post-stroke complications, especially pneumonia, which is an important determinant of patient mortality (46, 47). On the other hand, intracerebral hemorrhage (ICH) is often complicated by neurogenic pulmonary edema, a condition in which the systemic inflammatory response may play a crucial role (48). As such, the NLR encompasses not only the potential for secondary brain damage but also extracranial complications, thereby potentially exhibiting robust predictive capability in determining unfavorable outcomes.

Inflammatory processes rapidly ensue following the onset of a stroke event and possess the capability to exacerbate the damage within the hemorrhagic brain tissue (49). The neuroinflammatory response contributes crucially to brain cell demise, hematoma enlargement, edema development, and elevated intracranial pressure through the release of cytotoxic mediators, enhancement of capillary permeability, and facilitation of blood–brain barrier disruption (50–53). Therefore, most of the white cells infiltrating the brain are derived from peripheral blood, with neutrophils being the earliest recruited blood-derived cells. Injury to vulnerable regions of the central nervous system can enhance sympathetic nervous system or hypothalamic–pituitary–adrenal axis function, promoting peripheral blood lymphocyte apoptosis. Lymphocytes, being crucial components in cellular and humoral immune responses, when deficient can result in post-stroke immunosuppression and an increased vulnerability to infectious complications (54–56). Given the intimate connection between the immune system and the pathophysiological processes underlying intracerebral hemorrhage (ICH), it is unsurprising that comprehensive parameters reflecting immune responses triggered by the cerebral hematoma, both locally and systemically, may correlate with the clinical course of the disease. Indeed, clinical factors such as fever upon admission, elevated neutrophil counts, diminished lymphocyte levels, increased C-reactive protein, and heightened interleukin-6 concentrations have been independently linked to unfavorable prognoses in patients suffering from acute intracerebral hemorrhage (46, 47, 52, 57).

Consequently, NLR has emerged as a meaningful and widely accessible composite index, integrating information pertaining to both innate and adaptive immune responses (58). In the acute phase of ICH, neutrophils can serve as a marker for the inflammatory cascade reaction after hematoma and simultaneously reflect the possibility of secondary brain injury and susceptibility to post-stroke complications (neutrophilia and lymphopenia). As a result, the ratio of NLR has gained recognition as a significant and readily available comprehensive marker, combining insights into both the innate and adaptive arms of the immune system. Consequently, the NLR exhibits an independent association with prognosis, surpassing the predictive utility of total white blood cell count, neutrophil count, or lymphocyte count when considered individually (37). In addition, studies have shown that hyperglycemia may aggravate oxidative stress and inflammatory response, and thus may be combined with NLR to predict the clinical outcomes of ICH patients. Hyperglycemia increases ROS production by activating the NADPH oxidase pathway, and synergizes with ROS released by neutrophils to aggravate blood–brain barrier damage (59). Studies have shown that patients with NLR ≥ 7.5 and admission blood glucose >8.3 mmol/L have a 2.8-fold higher risk of symptomatic intracranial hemorrhage than those with a single abnormal indicator (*p* = 0.003) (59, 60). In addition, hyperglycemia-induced insulin resistance can inhibit lymphocyte proliferation and function, aggravate immune senescence, and make the NLR predictive efficacy of elderly ICH patients more significant (59). Studies have found that in patients aged ≥65 years, the sensitivity of NLR ≥ 7.5 combined with blood glucose >7.0 mmol/L in predicting mortality is 82% (AUC = 0.79) (59, 60). Therefore, in the future, more prospective studies on the use of NLR combined with known risk factors to predict the clinical outcomes of ICH patients can be carried out to build a more accurate prediction model to identify high-risk patients.

There are several limitations in this meta-analysis. First, most of the included studies were retrospective studies, and the control of confounding variables was insufficient, which resulted in a low overall credibility of the evidence quality of this study, and more prospective studies are needed to confirm it. Second, when conducting subgroup analysis, most studies were from Asia, there were few studies from Europe, and there were no studies from America and Africa, which may lead to selection bias, thus limiting the generalization of the conclusions of this study. Future prospective studies can consider adopting an international multicenter study design and include more patients from America and Africa to verify the universality of the conclusions of this study. The advantage of this meta-analysis is that it is the largest and most recent meta-analysis on NLR predicting ICH prognosis. Subgroup analysis determined the best population for NLR to predict ICH, and further verified the stability of NLR in predicting ICH prognosis. It showed that NLR can predict mortality, neurological outcome, and SAP incidence in ICH patients, suggesting that the clinical prediction model for ICH prognosis should consider NLR indicators and the best population.

## 5 Conclusion

This study found that NLR could significantly predict mortality, neurological function outcomes, and SAP in ICH. Considering that most studies in this meta-analysis were retrospective, with the majority from Asia, and the presence of potential selection bias and uncontrolled confounding factors, further large-scale prospective studies are needed to confirm the predictive value of NLR for ICH prognosis.

## Data Availability

The original contributions presented in the study are included in the article/Supplementary material, further inquiries can be directed to the corresponding author.
