# Supplementary material for: Neutrophil-to-lymphocyte ratio as a predictor of prognosis in patients with spontaneous intracerebral hemorrhage: a systematic review and meta-analysis
Source: Front Neurol. 2025 Mar 21;16:1553263. doi: 10.3389/fneur.2025.1553263 (PMC11968378; doi:10.3389/fneur.2025.1553263)
Supplement: Supplementary file 1 [file Data_Sheet_1.pdf]

Supplementary materials

Supplementary Table 1 The searching strategy

Databases	Searching strategy	Number of literature
Pubmed	(((("Neutrophils"[Mesh]) OR (((((((((((((Neutrophil) OR (Leukocytes, Polymorphonuclear)) OR (Leukocyte, Polymorphonuclear)) OR (Polymorphonuclear Leukocyte)) OR (Polymorphonuclear Leukocytes)) OR (Polymorphonuclear Neutrophils)) OR (Neutrophil, Polymorphonuclear)) OR (Polymorphonuclear Neutrophil)) OR (LE Cells)) OR (Cell, LE)) OR (LE Cell)) OR (Neutrophil Band Cells)) OR (Band Cell, Neutrophil)) OR (Neutrophil Band Cell))) AND (("Lymphocytes"[Mesh]) OR (((((Lymphocyte) OR (Lymphoid Cells)) OR (Cell, Lymphoid)) OR (Cells, Lymphoid)) OR (Lymphoid Cell)))) AND (("Intracranial Hemorrhages"[Mesh]) OR ((((((((((Hemorrhages, Intracranial) OR (Intracranial Hemorrhage)) OR (Hemorrhage, Intracrania)) OR (Posterior Fossa Hemorrhage)) OR (Hemorrhage, Posterior Fossa)) OR (Hemorrhages, Posterior Fossa)) OR (Posterior Fossa Hemorrhages)) OR (Brain Hemorrhage)) OR (Brain Hemorrhages)) OR (Hemorrhage, Brain)) OR (Hemorrhages, Brain)))) AND (ratio)	189
Embase	((NeutrophilsMesh or (Neutrophil or Leukocytes, Polymorphonuclear or Leukocyte, Polymorphonuclear or Polymorphonuclear Leukocyte or Polymorphonuclear Leukocytes or Polymorphonuclear Neutrophils or Neutrophil, Polymorphonuclear or Polymorphonuclear Neutrophil or LE Cells or Cell, LE or LE Cell or Neutrophil Band Cells or Band Cell, Neutrophil or Neutrophil Band Cell)) and (LymphocytesMesh or (Lymphocyte or Lymphoid Cells or Cell, Lymphoid or Cells, Lymphoid or Lymphoid Cell)) and (Intracranial HemorrhagesMesh or (Hemorrhages, Intracranial or Intracranial Hemorrhage or Hemorrhage, Intracrania or Posterior Fossa Hemorrhage or Hemorrhage, Posterior Fossa or Hemorrhages, Posterior Fossa or Posterior Fossa Hemorrhages or Brain Hemorrhage or Brain Hemorrhages or Hemorrhage, Brain or Hemorrhages, Brain)) and ratio).af.	407

Cochrane	((((NeutrophilsMesh) OR (((((((((((((Neutrophil) OR (Leukocytes, Polymorphonuclear)) OR (Leukocyte, Polymorphonuclear)) OR (Polymorphonuclear Leukocyte)) OR (Polymorphonuclear Leukocytes)) OR (Polymorphonuclear Neutrophils)) OR (Neutrophil, Polymorphonuclear)) OR (Polymorphonuclear Neutrophil)) OR (LE Cells)) OR (Cell, LE)) OR (LE Cell)) OR (Neutrophil Band Cells)) OR (Band Cell, Neutrophil)) OR (Neutrophil Band Cell))) AND ((LymphocytesMesh) OR (((((Lymphocyte) OR (Lymphoid Cells)) OR (Cell, Lymphoid)) OR (Cells, Lymphoid)) OR (Lymphoid Cell)))) AND ((Intracranial HemorrhagesMesh) OR (((((((((((Hemorrhages, Intracranial) OR (Intracranial Hemorrhage)) OR (Hemorrhage, Intracrania)) OR (Posterior Fossa Hemorrhage)) OR (Hemorrhage, Posterior Fossa)) OR (Hemorrhages, Posterior Fossa)) OR (Posterior Fossa Hemorrhages)) OR (Brain Hemorrhage)) OR (Brain Hemorrhages)) OR (Hemorrhage, Brain)) OR (Hemorrhages, Brain)))) AND (ratio)	3
Web of Science	((((NeutrophilsMesh) OR (((((((((((((Neutrophil) OR (Leukocytes, Polymorphonuclear)) OR (Leukocyte, Polymorphonuclear)) OR (Polymorphonuclear Leukocyte)) OR (Polymorphonuclear Leukocytes)) OR (Polymorphonuclear Neutrophils)) OR (Neutrophil, Polymorphonuclear)) OR (Polymorphonuclear Neutrophil)) OR (LE Cells)) OR (Cell, LE)) OR (LE Cell)) OR (Neutrophil Band Cells)) OR (Band Cell, Neutrophil)) OR (Neutrophil Band Cell))) AND ((LymphocytesMesh) OR (((((Lymphocyte) OR (Lymphoid Cells)) OR (Cell, Lymphoid)) OR (Cells, Lymphoid)) OR (Lymphoid Cell)))) AND ((Intracranial HemorrhagesMesh) OR (((((((((((Hemorrhages, Intracranial) OR (Intracranial Hemorrhage)) OR (Hemorrhage, Intracrania)) OR (Posterior Fossa Hemorrhage)) OR (Hemorrhage, Posterior Fossa)) OR (Hemorrhages, Posterior Fossa)) OR (Posterior Fossa Hemorrhages)) OR (Brain Hemorrhage)) OR (Brain Hemorrhages)) OR (Hemorrhage, Brain)) OR (Hemorrhages, Brain)))) AND (ratio)	136

Supplementary Table 2. Quality evaluation of the eligible studies with Newcastle–Ottawa scale

	Selection				Comparability		Outcome		
Study	Representative-ness	Selection of non-exposed	Ascertainment of exposure	Outcome not present at start	Comparability on most important	Comparability on other risk factors	Assessment of outcome	Long enough follow-up (median≥3	Adequacy (completeness) of follow-up

					factors			month)	
Tao, C. Y.2016-I[15]	*	*	*	*	*	*	*	*	*
Tao, C. Y.2016-II[31]	*	*	*	*	*	-	*	*	*
Wang, R. H.2023[29]	*	*	*	*	*	-	*	*	*
Menon, G2021[35]	*	*	*	*	-	-	*	*	*
Lin, M. Q.2023[36]	*	*	*	*	*	-	*	-	*
Pereira, M.2023[34]	*	*	*	*	*	-	*	-	*
Wang, F.2015[14]	*	*	*	*	*	-	*	-	*
Giede-Jeppe, A.2018[39]	*	*	*	*	*	-	*	*	*
Radu, R. A.2021[32]	*	*	*	*	*	*	*	-	*
Lattanzi, S.2018[38]	*	*	*	*	*	*	*	-	*
Wang, Z. G.2019[28]	*	*	*	*	*	-	*	-	*
Li, L.2022[37]	*	*	*	*	*	-	*	-	*
Lattanzi, S.2016[12]	*	*	*	*	*	*	*	*	*
Volbers, B.2018[30]	*	*	*	*	*	-	*	*	*
Zhang, F.2019-I[25]	*	*	*	*	*	-	*	*	*
Zhang, F.2019-II[26]	*	*	*	*	*	-	*	*	*
Zhao, Y.2022[22]	*	*	*	*	*	-	*	-	*
Qiu, W. J.2023[33]	*	*	*	*	*	-	*	*	*
Yang, W.2021[27]	*	*	*	*	*	-	*	*	*
Zhang, P.2020[24]	*	*	*	*	*	*	*	*	*
Zhao, Y.2023[23]	*	*	*	*	*	-	*	-	*

*indicates criterion met; - indicates significant of criterion not met.

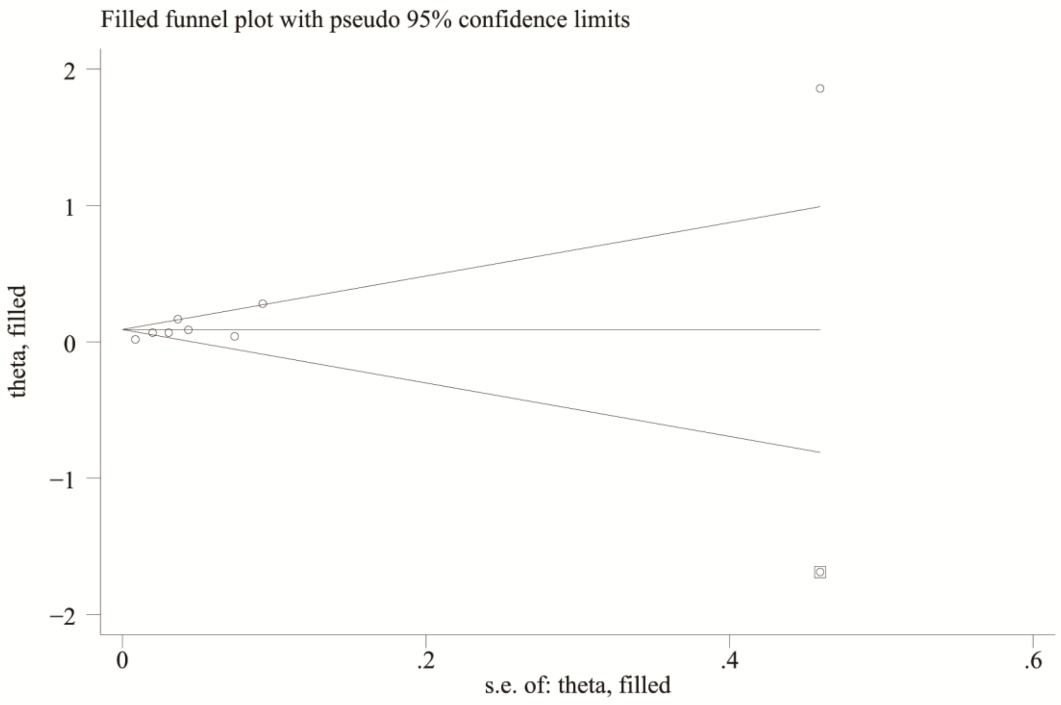

Supplementary Figure 1 Funnel plot of trim-and-fill method for predicting mortality using NLR

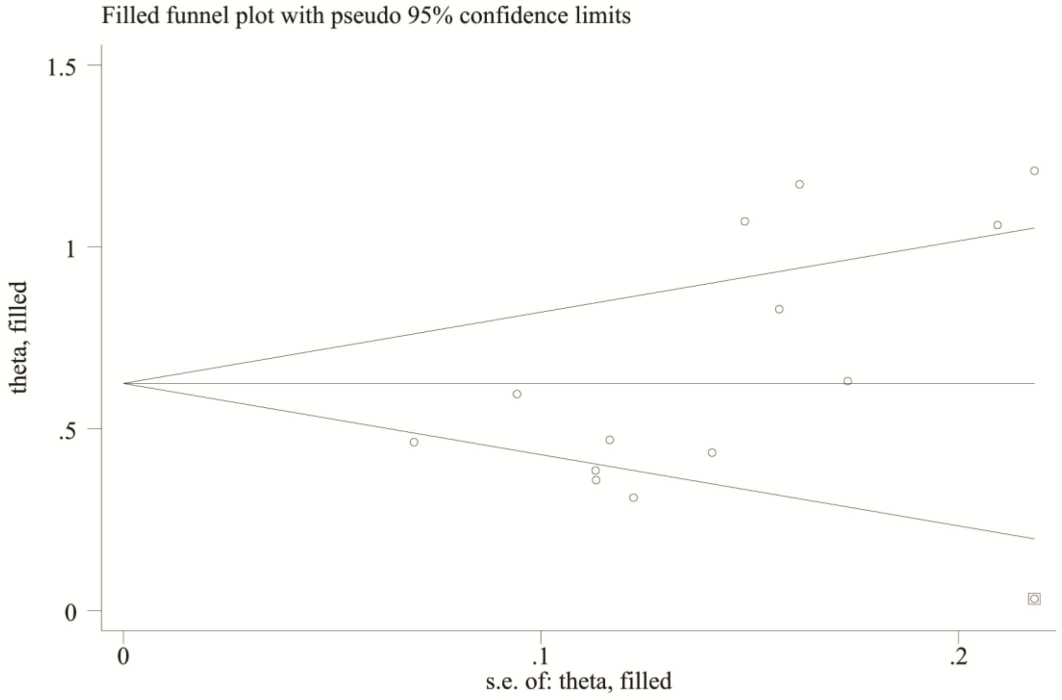

Supplementary Figure 2 Funnel plot of trim-and-fill method for predicting neurological function prognosis using NLR

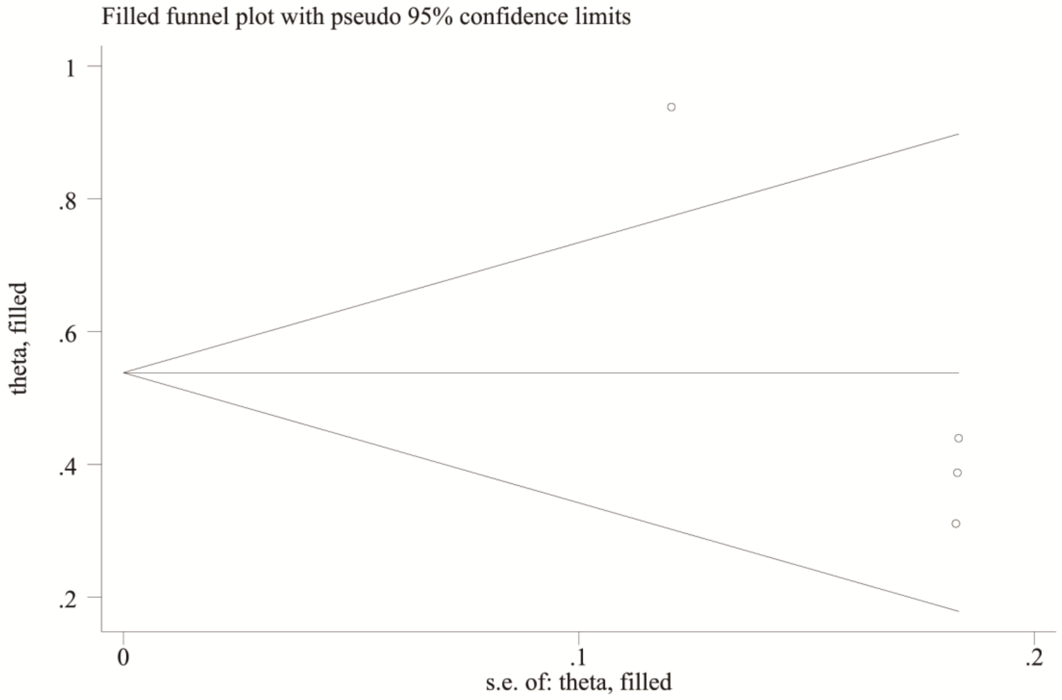

Supplementary Figure 3 Funnel plot of trim-and-fill method for predicting SAP using NLR

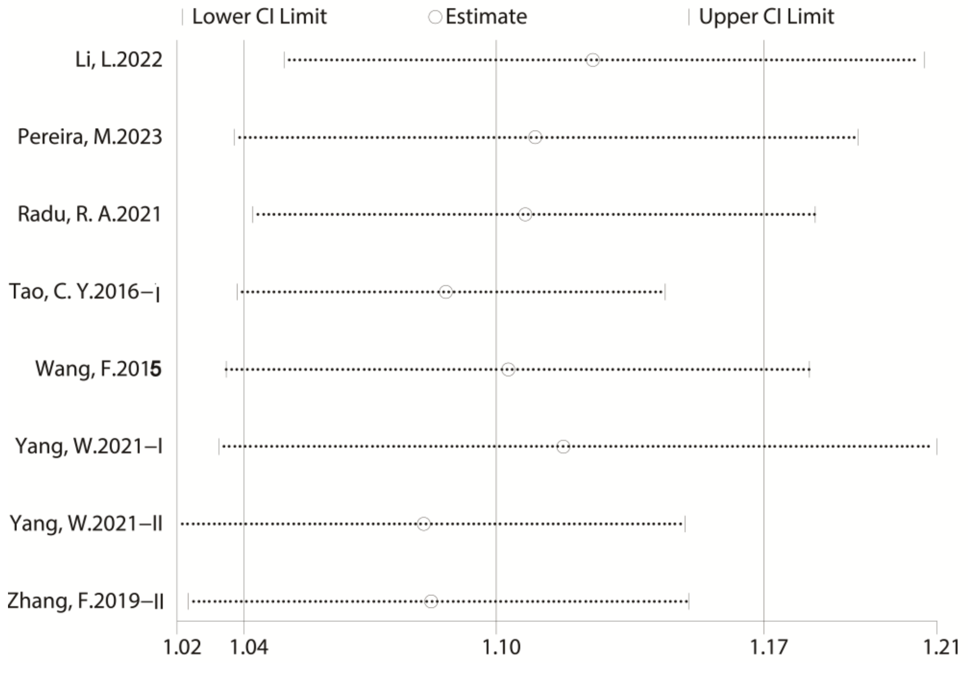

Supplementary Figure 4 Mortality sensitivity analysis (categorical variables)

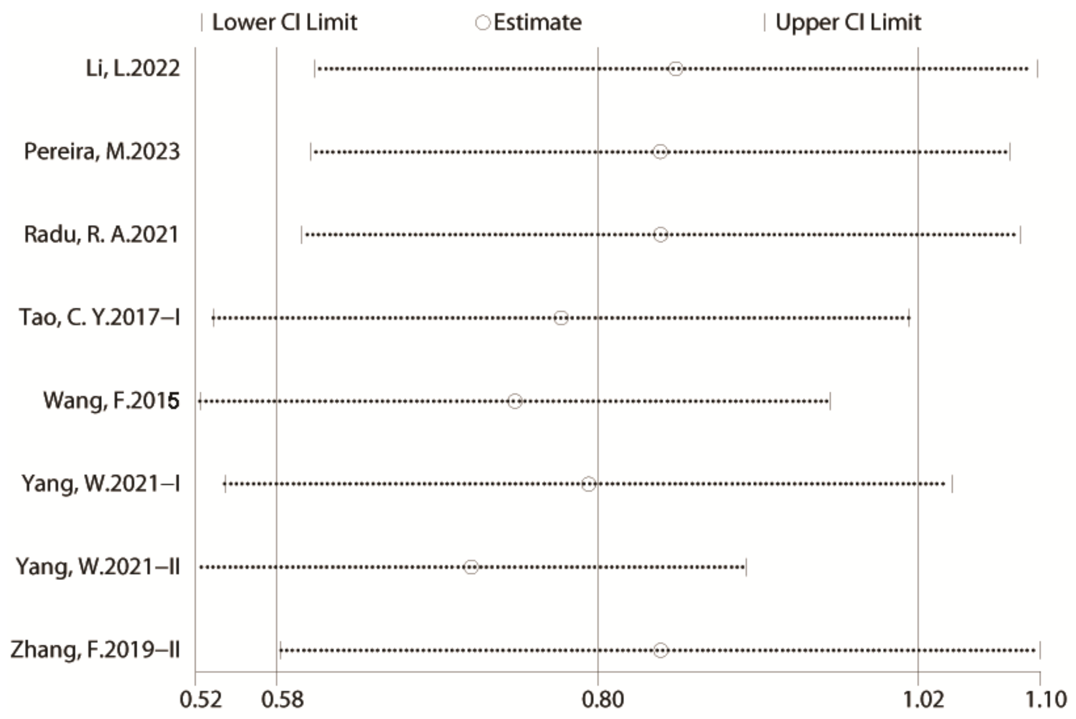

Supplementary Figure 5 Mortality sensitivity analysis (continuity variable)

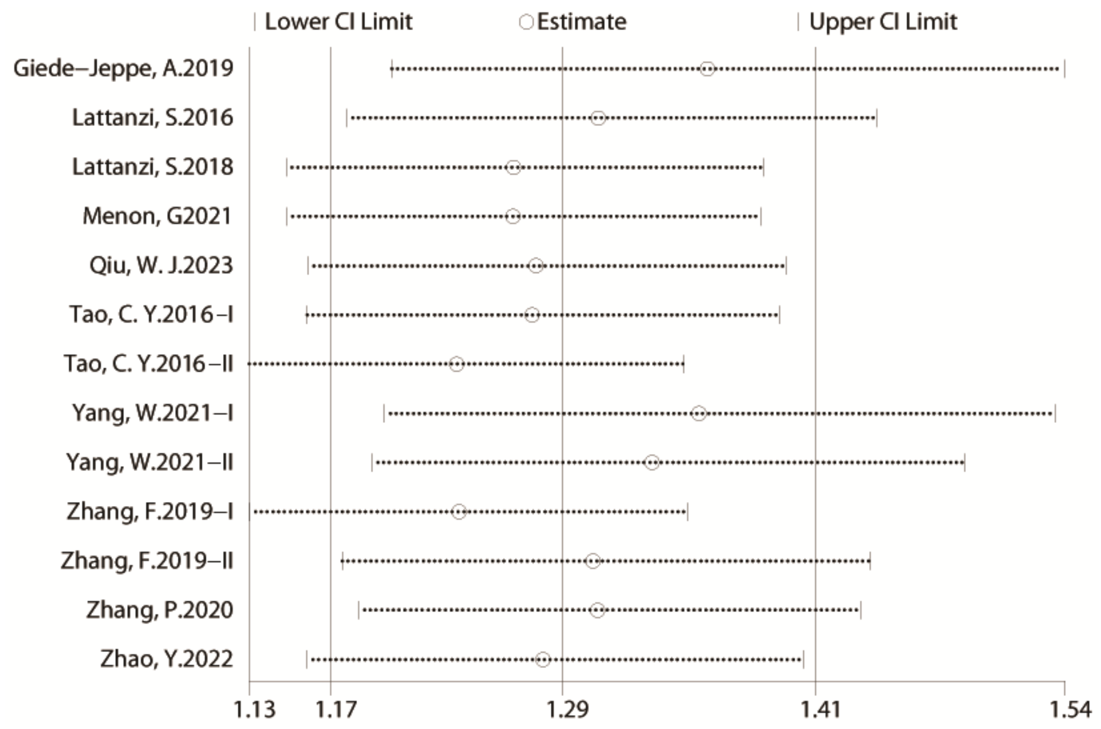

Supplementary Figure 6 Neural function sensitivity analysis (categorical variables)

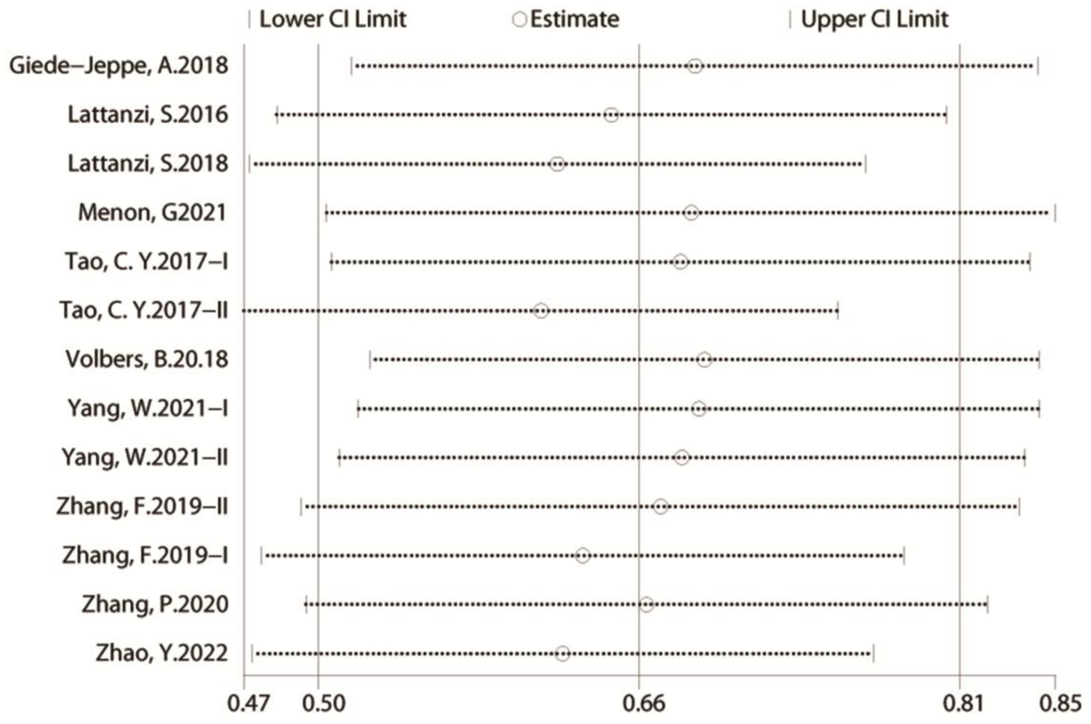

Supplementary Figure 7 Neural function sensitivity analysis (continuity variable)

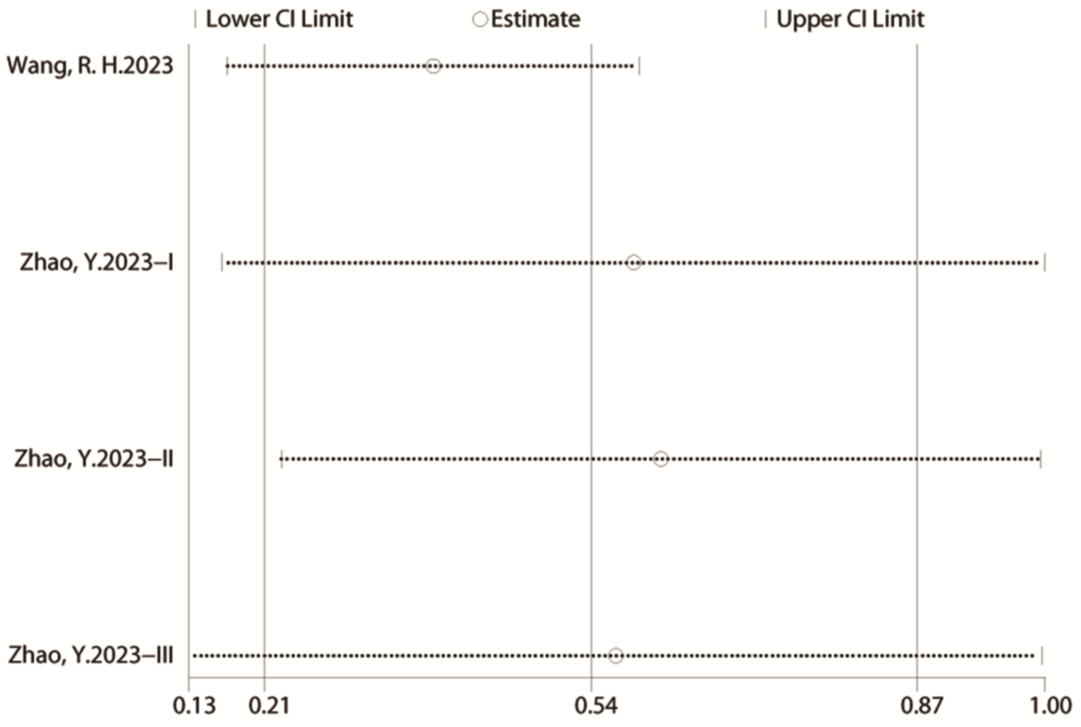

Supplementary Figure 8 SAP Sensitivity Analysis (Continuity variables)
